# Philadelphia Obstetrical Society

**Published:** 1890-08

**Authors:** 


### PHILADELPHIA OBSTETRICAL SOCIETY.

### Meeting of April 3, 1890.

From the *Annals of Gynecology and Pediatrics.*

[Extract.]

### PERFORATION OF THE UTERUS WITH A CURETTE — LAPARATOMY — RECOVERY.

Dr. Joseph Hoffman. — I was asked, the 25th of February, to see a patient in consultation, with a history of delayed miscarriage, of what was considered a six weeks' conception. I advised that the os should be dilated and the uterus curetted, to free it from the putrid product of conception. This was at once assented to, and the procedure initiated by the attending physician, a gentleman of no little experience in cases of the kind. The dilatation was done under ether with a Molesworth instrument, and the curetting attempted with the instrument here shown. There was no sign of any accident until shortly after the introduction of the curette and the removal of several fragments of partially decomposed placenta, when the operator noticed, as I myself had done, that the curette entered too far up for a pregnancy of so short a period. I said nothing, however, believing that an error had been made as to the duration of the pregnancy. At the operator's request, and seeing his alarm at the depth to which the curette penetrated, I took the instrument and carefully introducing it far up, directing it anteriorily against the abdominal wall, easily determined that it was in direct relation with it. At the operator's request, I continued the work of cleaning out the uterus, which was done as rapidly and carefully as possible, so as to avoid re-entering the abdominal cavity. When about to cease curetting, I noticed protruding slightly from the os what I at once concluded was omentum. This opinion was confirmed by gently drawing it down with a tenaculum.

I did this both with the idea of determining certainly that the wall of the uterus had been pierced through, and of temporarily tamponing the perforation with the omentum, to control hemorrhage, and, so far as possible, shut off the peritoneal cavity from the infection of the decomposing placenta. The woman was then put back to bed, and operation speedily arranged. Four hours afterward she was again etherized and the abdomen opened. The opening of the peritoneum was followed by the escape of a bloody fluid and some clots. The peritoneum was markedly injected, as was also its intestinal investment. In order to bring up the uterus so as to examine it for the rupture, the incision was enlarged to about four inches. The organ was found to be peculiarly softened, suggesting by its consistency a fatty kidney; there were present cracks in its anterior peritoneal covering, through which blood oozed. These may have been caused by the forcible extraction of the organ through the abdominal incision. The perforation at first did not come into view, but a little search and further extraction brought it to sight. It was situated upon the antero-lateral surface of the organ, a little below the middle. It was ragged, and of sufficient size to admit the end of a little finger. The rent was closed by interrupted silk sutures, and bled freely up to the tying of the last. Indeed, an additional one was introduced, more for the stoppage of hemorrhage than aught else. The abdomen was then flushed with hot water, and a drainage-tube introduced. Through this, fluid escaped rather profusely for three days, when a rubber tube was substituted. This acted efficiently save for one day, when it became choked with lymph. The tube was removed entirely the seventh day. A peritoneal fistula persisted for three days, when the wound closed fully, and she progressed rapidly to recovery. Owing to the length of the incision, the patient was kept in bed for three weeks. A stitch-hole abscess caused the only rise of temperature. At the present writing she is entirely comfortable, goes out daily for a ride or walk in fair weather, and, in all respects, may be considered well.

It is only fair to say here that the attending physician does not agree with me that the curette caused the perforation, but attributes it to the dilator. That there is some measure of probability that such is the case I can readily understand, though the location of the rupture, according to my opinion, was so far up as to be out of reach of the end of the dilator, and, therefore, could not have been caused by it. This question, however, is a matter of so little importance that it is not worth further consideration.

The individual value of the case here recorded rests upon the fact that in abortions a frequent necessity for curetting and dilating the uterus arises. It shows that in special cases the uterus may be pathologically softened, and that a very moderate force may cause the accident here noted. It needs no argument to show that in simple dilatation of the cervix rupture may also take place. One such case, followed by death in a short period, has lately come under my notice.

This fact will also explain not only deaths from forcible dilatation, but as well the cases of acute peritonitis sometimes following the operation, with tedious recovery and necessary subsequent pelvic operation.

Traumatisms in the removal of intrauterine fibroids by the spoon saw, or écraseur, or other means, may readily extend through the peritoneal coat of the organ, and speedily terminate fatally, if this fact is lost sight of. This is beyond doubt the cause of death of a majority of the patients lost by Baker Brown in his operations for uterine fibroids. Probably the most frequent cause of perforating wound of the uterus is the uterine sound. Fortunately the accident is rarely fatal, though it sometimes results in persistent utero-peritoneal fistula, which is abundantly proven by the cases of Sir J. Y. Simpson, as recorded by Mr. Tait. If one thing stands out more plainly than any other in this case, it is the value of early operation, if we would hope for favorable results in such or similar conditions or accidents. In another case of almost the same history, where the uterus was dilated and a filthy sponge tent introduced, and sepsis produced through a rupture of the uterus, operation was delayed until the abdomen was filled with pus, and of course the woman died. Those of us who believe in the necessity of early operation at once when diagnosis is made, need have no fear that our importunity will be patiently heard or prudently acted upon. There are, there must be, dawdlers and dalliers in surgery as elsewhere, who will wait for a puerperal rigor before they will operate; or, as they choose to express it, "run the risk of operation," and while they "stand lingering, shivering on the brink," the rigor comes, and, aided by their silly procrastination, kills the patient; then the case is reported as an operation in extremis, and the death excused for this reason.

Three such cases have come under my immediate notice within the last few weeks, and while such a condition of affairs exists it is our business and our duty to urge in season and out of it, the folly, the insanity, or, if you please, the professional shame, that such things should be. Here is no place for sentimental mewling about conservatism. Here is a common ground where "preservation" and "conversation" mean the same thing, and "conservatism" as a word should be dropped from our vocabulary as insane and senseless, a breath without an idea.

DISCUSSION.

Dr. William Goodell.—I should like to give my experience with the curette. While I congratulate Dr. Hoffman on his pluck and upon the result of his case, and while, with his view of the case, I think that he was justified in operating, yet I am disposed to think it was not necessary.

For instance, a lady patient of mine had a uterine hemorrhage after the age of 75 years. I removed with the curette, on various occasions, many papillary vegetations, evidently malignant. But, the last time I operated, the curette perforated the womb. This did not produce the slightest symptoms, and she died nine months afterward from the disease.

Dr. Taylor will recall an analogous case which we saw together. I was curetting malignant vegetations from the endometrium, when the instrument passed into the abdominal cavity, carrying, of course, with it cancerous juices, yet no appreciable results followed. While curetting a cancer of the cervix, I got into the abdominal cavity, and touched with my finger an ovary and a loop of bowel. Cancerous matter undoubtedly entered the cavity, but there was not the slightest septic infection.

I operated in another case of cancer by the high operation on the cervix, and in making the posterior incision I entered the abdominal cavity and exposed a bowel. I closed the opening by sutures, and not a bad symptom followed.

In yet another case, where I was searching for the cervix uteri, in a case of great sloughing of the bladder, womb, and soft parts, following labor, I cut into the peritoneal cavity. Unquestionably, urine passed in, yet this patient recovered after only a slight rise of temperature for two days. The cervix had probably sloughed away, and the uterine cavity most likely communicated directly with the bladder. But this I did not think of at the time. So, two weeks later, I made another effort to find and dissect out the cervix, and again, to my great surprise, got into the peritoneal cavity. As before, no harm followed this second misadventure.

Dr. M. Price.—A year ago I saw a case of criminal abortion. There was retained placenta and a septic condition. I removed the placenta, but the pelvic inflammatory trouble increased. I operated, and found a large pelvic abscess. Owing to the great prostration, I decided simply to drain. On the fifth or sixth day she died. At the *post-mortem* an opening large as the little finger was found. If the opening had been closed at the time of operation, the woman might have recovered.

Dr. Davis.—The case described by Dr. Hoffman is of interest to me, because I met with a similar case in hospital practice not long since. The patient had syphilis, and the fetus had died. After suffering from preliminary pains for several days, the woman came into labor at eight and a half months, with a temperature of 102°. After the expulsion of a macerated fetus, the placenta was retained. An effort to remove it by the hand resulted in getting away most of it, but a portion was firmly adherent. It was decided to allow it to remain, and to employ antiseptic treatment until it should be loosened. Thirty-six hours later, the patient's temperature rose. In my absence, the resident physician removed the remainder of the placenta with a small sharp curette, similar to that exhibited by Dr. Hoffman.

In making one especial effort with the curette, it seemed to penetrate farther than was expected, although no complaint of pain occurred. This was followed by a rapid and extreme fall in the temperature. The case subsequently died of puerperal sepsis, some two weeks afterward. At the *post-mortem* examination, the fundus was found in an encapsulated abscess, and at the fundus was an area of gangrenous muscular tissue, where the placenta had been adherent by syphilitic placentitis, and gangrene of the muscle had resulted. During this case, intra-uterine injections had been given, but had always returned promptly, and there was no evidence that the uterus had ever been perforated.

A method of treating retained placenta, when complicated by sepsis, which has recently been practised in Holland, is the vaginal extirpation of the uterus, and several successes by this method have been reported.

It is my own practice never to introduce into the uterus a sharp curette; the only one which I employ is the curette of Carl Braun, which is an instrument whose blade is as large as the thumb nail, having an edge no sharper than that of an ivory paper cutter; with this curette I have never seen injury inflicted.

Dr. William H. Parish.—I desire to say a few words in connection with the report of this case, inasmuch as it brings up a method of treatment of abortion which I consider extremely hazardous—that is, the use of the curette. If a curette is used, it should not be such an instrument as the one employed by Dr. Hoffman. In the "American System of Obstetrics," even Simon's scoop is figured as the instrument for the removal of the placenta. The use of that instrument is attended with very great danger. I have seen one case in consultation where, after a miscarriage of five months, the physician had used the latter instrument, and at the autopsy it was found that it had scraped deeply into the placental site, the normal roughness having been mistaken for partial attachment of an adherent placenta.

My own practice is the removal of the products of conception by the finger. This is not so difficult as it is thought to be by some. Some weeks ago, I saw, with Dr. O'Hara, a case of retained fetus at the fourth month, which had been dead for about a fortnight. I dilated with a tupelo tent for a few hours, and then with my finger removed the fetus and placenta through an os large enough to admit the use of only one finger. I first brought through the os a leg, which quickly separated from the body, as the fetus was putrescent. Then I secured the body, which separated at the neck. I then carried the finger into the brain cavity, removed the brain, made a crochet of my finger, and extracted the collapsed head. Finally, by combined manipulation, I removed the placenta with my finger, and washed out the uterus with solution of corrosive sublimate, 1 to 4,000, using a double-current tube. The patient recovered completely and rapidly. I have seen a number of such cases. I have never yet used the curette in abortion, and

I have never yet had a fatal case, though I have treated or participated in treatment of scores of cases.

There may be cases in which it would be proper to use the curette. Where there is recurring hemorrhage, with occasional somewhat dirty flow, and where it is known that no considerable portion of placenta remains, it would not be bad practice to use the *dull wire curette.* I have in the latter cases sometimes wrapped cotton around the dull curette, saturated this with a solution of bichloride of mercury, and then rubbed this over the intra-uterine surface, and detached fragments of the membrana decidua or minute portions of placental tissue, which have been productive of the hemorrhage. I then always wash out, or swab out, with an antiseptic. The use of the sharp curette to remove fragments of placenta is uncertain, unsafe, and unnecessary.

Dr. J. PRICE.—I agree with Dr. Parish in regard to the abuse of the curette. I do not see why the placenta cannot be delivered by the bimanual method, by the intelligent finger, and not by a metallic instrument. The reason of the failure is, that the physician tries to remove it with the patient buried in the bed. If he placed the patient on the table, he could clear the uterus without difficulty.

Dr. Goodell is reasoning about a different class of cases. To compare malignant cases with puerperal is scarcely fair. Surely, Dr. Goodell would not permit a quart of putrid septic *débris* to remain in the peritoneal cavity. The omentum was in the wound and it would have been difficult to reduce such a hernia by any other means than section. The septic condition could only be treated by drainage. Philadelphia is practically the only city in which cases of peritonitis are saved. Bantock states that he never saw a case of peritonitis operated on for that condition recover. In Germany they will not touch them.

The presence of healthy urine in the peritoneal cavity will do no mischief. Dr. Goodell has referred to the introduction of portions of cancer into the peritoneum. If I felt that I had contaminated the peritoneal cavity I should in all metro-peritoneal fractures at once close the opening, irrigate and drain the pelvis.

Dr. G. BETTON MASSEY.—The danger from the use of sounds might be lessened by the employment of one that is elastic. I have been using such a sound with much satisfaction. It is formed of a spiral of platinum, the flexible portion being two and a half inches long. The instrument is made of platinum, and this permits it to be heated red-hot to cleanse it, no other mode of cleansing being as efficient. It has sufficient elasticity to pass into the cavity, but yet will retain the curve imparted to it when withdrawn.

Dr. J. HOFFMAN.—It seems to me that in Dr. Goodell's case, where epitheliomatous matter was carried into the peritoneum, it would have been a good plan to open the abdomen to prevent contamination. The curette here shown has not a sharp edge; I can draw it forcibly over my hand without any bad result. I do not advocate the use of the curette where you can introduce the placental forceps. With them you can loosen and remove the retained placenta in a few minutes, and by revolving the forceps you can be sure that all is removed. Of all methods, I think that of gouging away the early placenta with the finger nail the most bungling and unskilful. It is painful and needlessly protracted.

In this case, when I opened the abdomen, there was a quart or more of fluid in the abdomen, and the temperature had begun to go up. I never expect to see a more typical irritation of peritonitis than that presented by both parietal and intestinal peritoneum.

